# Potassium Bromate Assay by Redox Titrimetry Using Arsenic Trioxide

**DOI:** 10.6028/jres.108.005

**Published:** 2003-02-01

**Authors:** Johanna M. Smeller, Stefan D. Leigh

**Affiliations:** National Institute of Standards and Technology, Gaithersburg, MD 20899

**Keywords:** arsenic trioxide, potassium bromate, redox titration

## Abstract

Bromate, a disinfectant, is one of the analytes of interest in wastewater analysis. Environmental laboratories have a regulatory need for their measurements to be traceable to NIST standards. Bromate is not currently certified as a NIST Standard Reference Material (SRM). Therefore, a traceable assay of potassium bromate (KBrO_3_) is needed.

KBrO_3_ was dissolved in water and assayed by redox titrimetry using arsenic trioxide (As_2_O_3_). A nominal (0.1 g) sample of As_2_O_3_ was dissolved in 10 mL of 5 mol/L sodium hydroxide. The solution was acidified with hydrochloric acid and about 95 % of the KBrO_3_ titrant was added gravimetrically. The end point was determined by addition of dilute (1:3) titrant using an automated titrator. The KBrO_3_ assay was determined to be 99.76 % ± 0.20 %. The expanded uncertainty considered the titrations of three independently prepared KBrO_3_ solutions.

## 1. Introduction

Bromate is an inorganic by-product of disinfectants. It is one of the analytes of interest in water supply analysis proficiency testing [1]. Environmental laboratories have a regulatory need to be traceable to NIST standards. Bromate is not currently certified as a NIST Standard Reference Material (SRM); thus, a traceable assay of potassium bromate (KBrO_3_) is needed.

Bromate is a strong oxidizing agent, which oxidizes iron (II), arsenic (III) and oxalate (C_2_O_4_^−2^) [2] and titrates directly with antimony (III), thallium (I), and hydrazine in acid medium [3]. Bromate may be used for the titration of mercury (I) and hexacyanoferrate (II) [2]. Bromate has been used for the determination of certain organic compounds, which undergo bromination of the aromatic rings, e.g., phenol and 8-quinolinol [2].

Many of the bromate titration methods use a visual end point detection. Some irreversible indicators used for bromate titrations are methyl red (color changes from red to yellow), methyl orange (color changes from red to yellow), and indigo sulfonic acid (color changes from blue to colorless) [2]. Reversible redox indicators that may be used are p-ethoxychrysoiden (color changes from red to colorless), quinoline yellow (color changes from yellow-green to colorless), and α-naphthoflavone (color changes from pale yellow to orange brown) [2]. Bromate may be titrated against standardized thiosulfate in acid medium with iodine and a catalyst (ammonium molybdate) [3,4,5,6]. Bromate mass fraction has been determined by titration with arsenious oxide in acid solution using an amperometric end point [7]. The method chosen here to assay the potassium bromate is the redox titration of bromate with arsenious oxide in acid medium [8,9,10], because of the availability of the primary standard, SRM 83d, Arsenic Trioxide Reductometric Standard, and the simplicity of the reaction.

## 2. Reagents

The following chemicals were used: Potassium bromate (KBrO_3_), ACS reagent; SRM 83d, Arsenic Trioxide (As_2_O_3_); 5 mol/L sodium hydroxide (NaOH) prepared from analytical reagent grade; 10 mol/L high-purity hydrochloric acid (HCl); and 1 % (mass fraction) methyl red indicator in ethanol (200 proof). All water used was 18 MΩ⋅cm. The KBrO_3_ was dried at 150 °C for 21 h, and the As_2_O_3_ was dried at 110 °C for 12 h. Both salts were stored over anhydrous magnesium perchlorate in a desiccator.

## 3. Procedure

Three solutions were prepared from the dried KBrO_3_ to a nominal mass fraction of 0.012 g/g. Each solution was titrated on a separate day. The assay procedure [8,9,10] was a redox titration in which As_2_O_3_ was titrated with potassium bromate according to Eq. (1) and Eq. (2).

$$3{\text{As}}_{2}O_{3}+2{\text{KBrO}}_{3}+9H_{2}O\to 6H_{3}{\text{AsO}}_{4}+2\text{KBr}$$ (1)

$${{\text{BrO}}_{3}}^{-}+5{\text{Br}}^{-}+6H^{+}\to 3{\text{Br}}_{2}+3H_{2}O.$$ (2)

According to Eq. (2), after all the As_2_O_3_ has been consumed, the end point (first appearance of free bromine) is detected by irreversible decolorization of the indicator and/or change in potential.

A nominal 0.1 g sample of As_2_O_3_ was weighed (± 0.00001 g) in a platinum boat. After transferring the sample to a 150 mL beaker, 10 mL of 5 mol/L NaOH was added. The concentration of NaOH is important to insure complete dissolution. It takes about 5 min to 10 min for the As_2_O_3_ to dissolve, and difficulty in dissolution occurs with more dilute NaOH. A magnetic stir bar, 50 mL of water, and 10 mL of 10 mol/L HCl were added to the solution. The resulting acidic medium is required for the titration method. The indicator, two drops of methyl red indicator, was added just before the start of the titration. At the end point, the indicator turns from red to colorless.

**Fig. 1:**
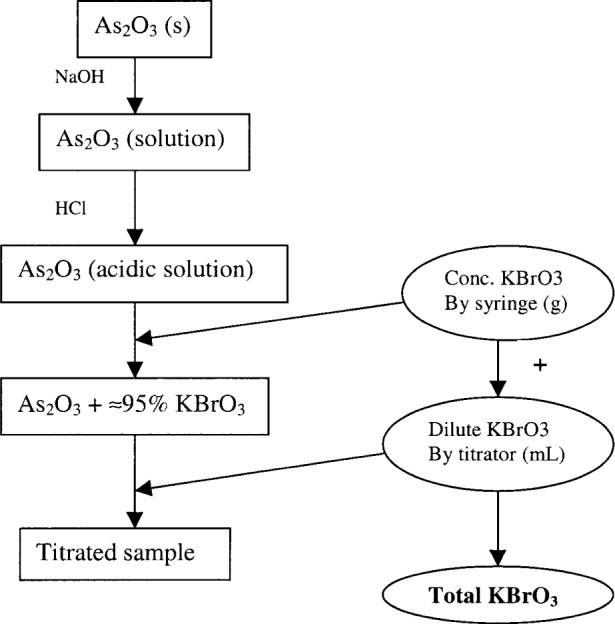
Flow diagram of the KBrO_3_ titrant additions.

The flow diagram (Fig. 1) illustrates the KBrO_3_ titrant additions.

Approximately 95 % of the KBrO_3_ titrant (gravimetric KBrO_3_) was added gravimetrically to the solution from a weighed (± 0.00001 g) plastic 5 mL or 10 mL syringe. This initial titrant addition (gravimetric KBrO_3_) is added quickly with visual help from the indicator change.

The remainder of the KBrO_3_ (volumetric KBrO_3_), about 0.4 mL of a more dilute solution with a nominal dilution factor of three, was titrated volumetrically to a potentiometric end point using an automated titrator. A visual end point from the indicator was also observed at this time. A combination platinum electrode (Schott Blue line 31 RX)^1^ was immersed in the solution on a sample changer and the titrant (dilute KBrO_3_) was added from a 10 mL buret of an automated titrator. As the solution was mixed by the rotating stir bar, the automated titrator added equal-volume (0.006 mL) increments of dilute KBrO_3_ titrant. Data stored included the volume of titrant added, *V*, with a corresponding measured potential, *E*, and numerical estimates of the first derivative (d*E*/d*V*). The end point was determined as the maximum of this first derivative. The amount of dilute KBrO_3_ added to reach the end point was the volumetric KBrO_3_. At least two blanks (reagents only, omitting As_2_O_3_) were titrated volumetrically with the dilute KBrO_3_ titrant each day.

The amount of gravimetric KBrO_3_ (g) and volumetric KBrO_3_ (mL) were added to calculate the titrant (total KBrO_3_) using Eq. (3) as follows:

$$m_{\text{total titrant}}=\left(m_{{\text{conc KBrO}}_{3}}+\frac{\rho (V_{{\text{dil KBrO}}_{3}}-V_{\text{blank}})}{DF}\right)$$ (3)

where

*m*_total titrant_ = mass of total KBrO_3_ (g) at the end point
$m_{{\text{conc KBrO}}_{3}}$ = mass of concentrated KBrO_3_ solution (gravimetric KBrO_3_) (g)
*ρ* = density of dilute KBrO_3_ solution (g/mL)
$V_{{\text{dil KBrO}}_{3}}$ = volume of dilute KBrO_3_ solution (mL)
*V*_blank_ = volume of dilute KBrO_3_ solution titrated for the blank (mL)
*DF* = dilution factor.

According to Eq. (4) below, the mass fraction (*w*), in %, of KBrO_3_ was calculated as the ratio of the KBrO_3_ (g/g) from the titration with As_2_O_3_ (1st factor) to the KBrO_3_ (g/g) from the preparation of the gravimetric solution (2nd factor) as follows:

$$w_{{\text{KBrO}}_{3}}=\left[\frac{m_{{\text{As}}_{2}O_{3}}w_{{\text{As}}_{2}O_{3}}2M_{{\text{KBrO}}_{3}}}{m_{\text{total titrand}}3M_{{\text{As}}_{2}O_{3}}}\right]\left[\frac{m_{{\text{grav KBrO}}_{3}\text{soln}}}{m_{{\text{grav KBrO}}_{3}\text{salt}}}\right]\times 100$$ (4)

where

$w_{{\text{KBrO}}_{3}}$ = mass fraction of KBrO_3_ (%)
$m_{{\text{As}}_{2}O_{3}}$ = mass of As_2_O_3_ (g)
$w_{{\text{As}}_{2}O_{3}}$ = mass fraction of As_2_O_3_ (g/g)
$M_{{\text{KBrO}}_{3}}$ = molecular weight of KBrO_3_ (g/mol)
$M_{{\text{As}}_{2}O_{3}}$ = molecular weight of As_2_O_3_ (g/mol)
*m*_total titrant_ = mass of total KBrO_3_ (g)
$m_{{\text{grav KBrO}}_{3}\;\text{soln}}$ = mass of KBrO_3_ gravimetric solution prepared from KBrO_3_ salt(g)
$m_{{\text{grav KBrO}}_{3}\;\text{salt}}$ = mass of KBrO_3_ (salt) for preparation of gravimetric solution (g).

The molecular weights (relative molecular masses) of KBrO_3_ and As_2_O_3_ are 167.001 g/mol and 197.8412 g/mol, respectively [11]. The mass measurements were corrected for air buoyancy. The densities of the dilute and concentrated KBrO_3_ solutions were determined. Corrections for air buoyancy were calculated based on densities [12] of 3.27 g/mL for KBrO_3_, 3.738 g/mL for As_2_O_3_, 0.00117 g/mL for air, and 8.0 g/mL for the stainless steel calibration weights in the microbalance [13].

## 4. Purity Analysis of KBrO_3_

A potassium bromate sample was analyzed by glow discharge mass spectrometry (GDMS) [14]. Among the element impurities found were arsenic and chlorine, present at 1 *µ*g/g and 10 *µ*g/g, respectively. Assuming the worst situation that all arsenic is present as As (III), and all chlorine as Cl (V), the maximum relative effects on the KBrO_3_ assay (mass fraction, %) of these two impurities are no greater than 0.001 % and 0.005 %, respectively, which is insignificant compared to the final expanded uncertainty (0.20 %) of the KBrO_3_ assay (mass fraction, %). The arsenic impurity is probably present as As (V), since As(III), if present, would be oxidized to As (V) by the bromate matrix. However, to estimate the worst possible effect, arsenic (determined by GDMS) is assumed to be As (III). No correction or further consideration regarding the GDMS analysis is given.

## 5. Results and Discussion

The recommended mass fraction value for KBrO_3_ and its uncertainty are summarized in Table 1. There is a difference among the titration results of the three solutions. The recommended value represents the combined mean mass fractions of the KBrO_3_ in solutions 1, 2, and 3. The uncertainty assigned to the recommended value is calculated by combining the uncertainties of the measurements of KBrO_3_ in the three solutions [15]. The resulting expanded uncertainty makes use of both within and between estimates of uncertainty. The within measurement uncertainty is calculated according to Eq. (5).

$$u_{\text{within}}=\frac{\sqrt{u_{1}^{2}+u_{2}^{2}+u_{3}^{2}}}{3}$$ (5)

where

*u*_within_ = within measurement uncertainty
*u*_1_ = combined uncertainty (*u*_c_) of solution 1
*u*_2_ = combined uncertainty (*u*_c_) of solution 2
*u*_3_ = combined uncertainty (*u*_c_) of solution 3.

The between measurement uncertainty component is determined according to Eq. (6).

$$u_{\text{between}}=\frac{\left|range\right|}{\sqrt{12}}$$ (6)

where

*u*_between_ = between measurement uncertainty
|*range*| = absolute value of the difference between the maximum mean value for a solution (2) and the minimum mean value for a solution (3).

The expanded uncertainty is found according to Eq. (7) using a coverage factor of 2 [15].

$$U=2^{*}\sqrt{{u_{\text{within}}}^{2}+{u_{\text{between}}}^{2}}.$$ (7)

Summaries of results for solutions 1, 2, and 3 are presented in Table 2. Uncertainties were determined using the ISO Guidelines [16]. The individual components of uncertainty (Type A and Type B) are listed in Table 3 for solution 1. The *u*_i_ represent the standard uncertainties associated with each of the uncertainty components, and the *c*_i_ represent the associated sensitivity coefficients [17]. Since the Type B uncertainty components for each solution are similar, only the uncertainty components of solution 1 are listed in Table 3. Comparisons of the individual uncertainty components are discussed later. Type A uncertainties are calculated from the standard deviations of the mean. Type A uncertainties represent the random variation in the following measurands: titration of KBrO_3_, titration of blanks, density, and the assay of As_2_O_3_ [18]. The combined Type A uncertainty is calculated using the root-sum-of-square (RSS).

The combined Type B uncertainty is calculated in a manner similar to that used to calculate the Type A uncertainty. The components of Type B uncertainty include the following: mass of As_2_O_3_, molecular weight of both As_2_O_3_ and KBrO_3_, mass of concentrated KBrO_3_ solution (titrant), volume of dilute KBrO_3_ solution, dilution factor of the dilute titrant (KBrO_3_ solution), mass of concentrated KBrO_3_ solution, mass of KBrO_3_ salt used to prepare the concentrated KBrO_3_ solution, and the mass of the concentrated KBrO_3_ solution.

The uncertainty of the dilution factor is calculated by combining the uncertainties of the two mass measurements used to prepare the dilute KBrO_3_ solution. A standard uncertainty of 30 *µ*g for each mass measurement with a 10 *µ*g resolution balance is estimated. The uncertainty of the mass of concentrated KBrO_3_ solution (titrant) is 100 *µ*g. This includes the uncertainties associated with the mass measurement of the filled syringe in a beaker, drift, and possible evaporation. It is calculated as the sum in quadrature of the uncertainty of the syringe before and after delivery of the titrant, and equals 141 *µ*g. Because the actual mass value is most likely near the center of this range, the uncertainty distribution is best modeled as a triangular distribution. The standard uncertainty is then 58 *µ*g $\left(141\mu g/\sqrt{6}\right)$. The mass measurement uncertainty of As_2_O_3_ is estimated to be 60 *µ*g. Its uncertainty is calculated as the sum in quadrature of the uncertainty of each mass measurement (As_2_O_3_ was weighed by difference) and equals 85 *µ*g. The corresponding standard uncertainty, using a triangular distribution, is 35 *µ*g $\left(85\mu g/\sqrt{6}\right)$.

To calculate the uncertainty of the volume of dilute KBrO_3_ solution, the uncertainty in the accuracy of the buret and the uncertainty associated with the volume additions from the titrator are combined in quadrature. The uncertainty in the accuracy of the 10 mL buret, according to manufacturer’s specification, is 0.15 % of the volume of dilute KBrO_3_ solution added (about 0.4 mL). Assuming a uniformly probable distribution for buret error, this value is converted to a standard uncertainty by division by $\sqrt{3}$. The volume of dilute KBrO_3_ solution additions from the titrator was 0.006 mL for solutions 2 and 3, and 0.01 mL for solution 1. Uncertainties for volume increments were computed as standard errors for assumed underlying triangular distributions ( $0.006\;\text{mL}/\sqrt{6}$ for solutions 2 and 3, and $0.01\;\text{mL}/\sqrt{6}$ for solution 1). The standard uncertainty of the volume of dilute KBrO_3_ was larger for solution 1 than for solutions 2 and 3.

The uncertainties in the molecular weight of both As_2_O_3_ and KBrO_3_ are calculated from the recommended uncertainties in the IUPAC assigned relative atomic masses [11] of the elements (As, O, K, Br) combined in quadrature. The corresponding standard uncertainty was calculated by dividing the IUPAC recommended uncertainty (99.7 % confidence interval) by 3. This estimation was based on interpretation by the NIST Statistical Engineering Division [19] of the language used in the IUPAC explanation [20].

**Fig. 2:**
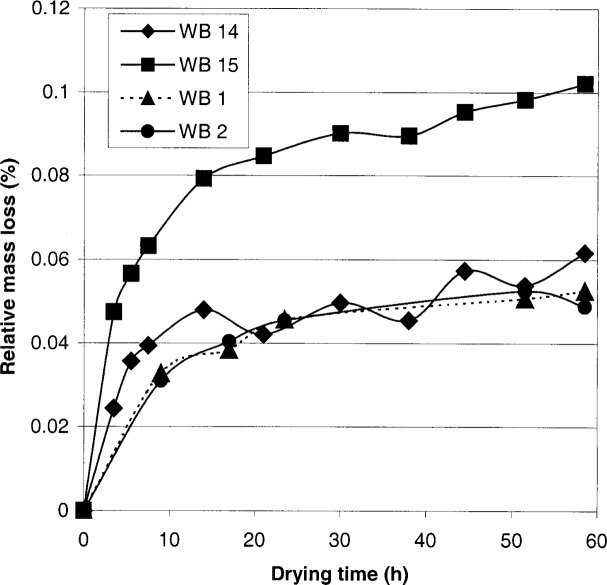
Relative mass loss of potassium bromate salt on drying vs drying time.

The uncertainty of the mass of KBrO_3_ salt used to prepare the concentrated KBrO_3_ solution was calculated in a different way than the other mass measurements. The mass of KBrO_3_ salt was measured at the end of a drying study (about 50 h drying time). In Fig. 2, the loss of mass of the KBrO_3_ salt on drying is plotted versus the drying time (h). The WB plot symbol identifies the weighing bottle for each sample and the ordinate identifies its corresponding mass loss. The four samples, taken from one bottle of KBrO_3_, were dried, and then used in the solution preparation for the samples to be titrated. Between 80 % and 90 % of the total mass loss is observed after 21 h. We have recommended a drying time of 24 h at 150 °C for KBrO_3_, unless this mass loss becomes a significant uncertainty component. Thus, the uncertainty of the mass of KBrO_3_ salt for each solution (solution 1, 2, and 3) is calculated to account for the difference between the mass loss at about 21 h of drying and the average mass loss at about 50 h. The uncertainty applies to the specific mass loss differences of a specific weighing bottle and the solution (solution 1, 2, and 3) that was prepared.

The uncertainty of the mass of the concentrated KBrO_3_ solution (preparation of solutions 1, 2, and 3) with a 1 mg resolution balance is 0.002 g. Assuming a rectangular distribution for the error in weighing $\left(0.002/\sqrt{3}\right)$ and considering that the mass of the concentrated KBrO_3_ solution was determined from two mass measurements (multiplied by $\sqrt{2}$) the standard uncertainty is 0.00163 g.

The most significant sources of uncertainty are the following: measurement replication of the titration of KBrO_3_, mass of As_2_O_3_, volume of dilute KBrO_3_ solution and, to a lesser extent, mass of KBrO_3_ salt. Generally, the Type A uncertainty varied the most. The uncertainty associated with measurement replication of solution 3 was greater than the measurement replication uncertainties of solution 1 (Table 3) and solution 2 because the uncertainties of the mass fractions of the titrant (KBrO_3_) and dilute titrant were greater for solution 3. The combined Type A uncertainty for solution 3 was 2.3 times greater than its combined Type B uncertainty. The uncertainty associated with measurement replication of solution 2 was the lowest. The combined Type B uncertainty for solution 2 was 2.0 times greater than its combined Type A uncertainty. Better measurement agreement across replications might have been obtained with solution 1 if the automated titrator had added dilute titrant in smaller increments (0.006 mL instead of 0.01 mL). The Type B uncertainties for all 3 solutions were similar. The uncertainty of the mass of As_2_O_3_ is greater than the other mass measurements because of the small sample mass (0.1 g). The small mass is important to insure complete dissolution. However, the use of a microbalance with better than 10 *µ*g resolution might improve this measurement. The uncertainty of the volume of dilute KBrO_3_ might be decreased by smaller volume increments of the automated titrator, and/or a larger dilution factor of the dilute titrant.

Individual titration assay results for solutions 1, 2, and 3 are listed in Table 4.

## Figures and Tables

**Table 1:** Summary of results for titrimetric assay of potassium bromate

	Solution 1	Solution 2	Solution 3	Combined
Determined value^a^ mass fraction (%)	99.796	99.900	99.586	99.761
Within component	0.063^b^	0.041^b^	0.107^b^	0.044
Between component				0.091
Combined uncertainty (*u*_c_)				0.101
Expanded uncertainty (*U*)^c^				0.201
Recommended value^a^, ^c^				99.76 ± 0.20

a Buoyancy corrected.

b Table 2.

c [15]; *k* = 2.

**Table 2:** Summary of results for titration assay of KBrO_3_, solutions 1, 2, 3

Potassium bromate	Solution 1	Mass fraction (%) Solution 2	Solution 3
Measured value^a^	99.796	99.900	99.586
Uncertainties			
Type A (*c*_i_*u*_i_)	0.045^b^	0.018^b^	0.098^b^
Type B (*c*_i_*u*_i_)	0.044	0.037	0.043
Combined uncertainty (*u*_c_)	0.063	0.041	0.107

a Buoyancy corrected.

b *n* = 12 measurements.

**Table 3:** Components of uncertainty for potassium bromate, solution 1

Potassium bromate mass fraction (%) for solution 1						
Type A						
Source	*u*_i_	units	*c*_i_	units	*c*_i_ *u*_i_	DF
Titration measurement replication	4.48E-04	g/g	99.8	1	4.47E-02	11
Mass fraction As_2_O_3_	1.36E-05	g/g	99.8	1	1.36E-03	11
Density of dilute KBrO_3_	3.72E-06	g/mL	−2.88	mL/g	−1.07E-05	4
Blank	1.00E-03	mL	6.93	g/gmL	6.93E-03	1
Combined type A uncertainty					0.0453	

Type B						
Source	*u*_i_	Units	*c*_i_	units	*c*_i_ *u*_i_	DF

Mass As_2_O_3_	3.46E-05	g	951	1/g	3.29E-02	∞
Molecular weight As_2_O_3_	3.00E-04	g/mol	−0.504	mol/g	−1.51E-04	∞
Molecular weight KBrO_3_	4.50E-04	g/mol	0.598	mol/g	2.69E-04	∞
Mass KBrO_3_	5.80E-05	g	−19.0	1/g	−1.10E-03	∞
Volume dilute KBrO_3_	4.10E-03	mL	−6.93	g/gmL	−2.84E-02	∞
Dilution factor	3.15E-07	g/g	1.05	1	3.30E-07	∞
Mass KBrO_3_ salt	4.69E-04	G	−19.1	1/g	−8.96E-03	∞
Mass KBrO_3_ solution	1.63E-03	G	0.216	1/g	3.52E-04	∞
Combined type B uncertainty					0.0444	
Effective degrees of freedom >30						

**Table 4:** Individual results for titration assay of KBrO_3_^a^

Potassium bromate mass fraction (%)		
Solution 1	Solution 2	Solution 3
99.821	99.785	99.156
99.521	99.886	99.152
99.598	99.892	99.258
99.680	99.820	99.242
100.027	99.884	99.230
99.726	99.931	99.826
99.767	99.879	99.849
99.836	99.914	99.931
99.759	99.901	99.796
99.895	99.958	99.885
99.898	99.989	99.872
100.027	99.962	99.838

a Buoyancy corrected.

## Footnotes

1 Certain commercial equipment, instruments, or materials are identified in this paper to foster understanding. Such identification does not imply recommendation or endorsement by the National Institute of Standards and Technology, nor does it imply that the materials or equipment identified are necessarily the best available for the purpose.
